# Exploring the Antimicrobial, Anticancer, and Apoptosis Inducing Ability of Biofabricated Silver Nanoparticles Using *Lagerstroemia speciosa* Flower Buds against the Human Osteosarcoma (MG-63) Cell Line via Flow Cytometry

**DOI:** 10.3390/bioengineering10070821

**Published:** 2023-07-10

**Authors:** Kariyellappa Nagaraja Shashiraj, Anil Hugar, Raju Suresh Kumar, Muthuraj Rudrappa, Meghashyama Prabhakara Bhat, Abdulrahman I. Almansour, Karthikeyan Perumal, Sreenivasa Nayaka

**Affiliations:** 1 P.G. Department of Studies in Botany, Karnatak University, Dharwad 580003, Karnataka, India; rajscbz@gmail.com (K.N.S.); anilh5157@gmail.com (A.H.); rmuthuraj20@gmail.com (M.R.); meghubhat09@gmail.com (M.P.B.); 2 Department of Chemistry, College of Science, King Saud University, Riyadh 11451, Saudi Arabia; sraju@ksu.edu.sa (R.S.K.); almansor@ksu.edu.sa (A.I.A.); 3 Department of Chemistry and Biochemistry, The Ohio State University, 151 W. Woodruff Ave, Columbus, OH 43210, USA; pkarthikjaya@gmail.com

**Keywords:** *Lagerstroemia speciosa* flower bud, bio-fabricated synthesis, silver nanoparticles, MTT assay, MG-63 cancer cell line, apoptosis/necrosis, flow cytometry

## Abstract

Biosynthesized nano-composites, such as silver nanoparticles (AgNPs), can be engineered to function as smart nano-biomedicine platforms for the detection and management of diverse ailments, such as infectious diseases and cancer. This study determined the eco-friendly fabrication of silver nanoparticles using *Lagerstroemia speciosa* (L.) Pers. flower buds and their efficacy against antimicrobial and anticancer activities. The UV-Visible spectrum was found at 413 nm showing a typical resonance spectrum for *L. speciosa* flower bud extract-assisted silver nanoparticles (Ls-AgNPs). Fourier transform infrared analysis revealed the presence of amines, halides, and halogen compounds, which were involved in the reduction and capping agent of AgNP formation. X-ray diffraction analysis revealed the face-centered cubic crystals of NPs. Energy dispersive X-ray verified the weight of 39.80% of silver (Ag), TEM analysis revealed the particles were spherical with a 10.27 to 62.5 nm range, and dynamic light scattering recorded the average particle size around 58.5 nm. Zeta potential showed a significant value at −39.4 mV, and finally, thermo-gravimetric analysis reported higher thermal stability of Ls-AgNPs. Further, the obtained Ls-AgNPs displayed good antimicrobial activity against clinical pathogens. In addition, a dose-dependent decrease in the anticancer activity by MTT assay on the osteosarcoma (MG-63) cell line showed a decrease in the cell viability with increasing in the concentration of Ls-AgNPs with an IC_50_ value of 37.57 µg/mL. Subsequently, an apoptotic/necrosis study was conducted with the help of Annexin-V/PI assay, and the results indicated a significant rise in early and late apoptosis cell populations. Therefore, green synthesized Ls-AgNPs were found to have potent antimicrobial and anticancer properties making them fascinating choices for future bio-medical implementations.

## 1. Introduction

Nanotechnology research will be at the forefront in the 21st century; it is a novel and developing technology with broad applications. It includes synthesizing new materials and their use in various fields with dimensions ranging from 1 to 100 nanometers, which could significantly change their physico-chemical properties. Several materials are used to make nanoparticles, and the chemical composition, size, and form can affect the particle’s characteristics [1]. The use of nanoparticles (NPs) is expanding in several fields, including environmental science and engineering, biomedical devices, electronics, pharmaceuticals, cosmetics, textiles, food processing industries, medicine, cancer therapy, and nano-medicine. New nanoparticle applications are likely to be discovered as research continues, leading to more advancements in science and technology [2,3]. Several metal and metal oxide nanoparticles were synthesized using various protocols, including palladium, selenium, zinc oxide, copper oxide, gold, and silver. In addition to other types of metal nanoparticles, silver nanoparticles (AgNPs) have demonstrated significant efficacy in promoting wound healing as a result of their inherent therapeutic properties [4].

Utilizing plant extracts to synthesize silver nanoparticles is a highly efficient, economically viable, and bio-sustainable approach that circumvents hazardous chemicals. In recent years, numerous bio-sustainable methods for the expeditious production of silver nanoparticles have been documented, which employ aqueous extracts of various plant components, such as leaves, bark, flowers, and roots [5]. The use of plant extracts for the synthesis of AgNPs conveys various benefits in comparison to the implementation of chemical, physical, and microbial approaches. The utilization of plant extracts obviates the need for the use of hazardous reducing and capping agents, high temperature and radiation, expensive microbial growth media, and microbial strains for the production of nanoparticles [6]. Additionally, phyto-mediated nanoparticles are typically biocompatible and biodegradable, making them safe to use in many applications [7]. The potential applications of phyto-synthesized AgNPs have been investigated in biomedical diagnostics, including their use as antibacterial, anticancer agents, and anti-inflammatory activities [8,9].

*Lagerstroemia speciosa* (L.) Pers. is a flowering tree in the Lythraceae family. It is indigenous to Southeast Asia, primarily the Philippines, but has been introduced and produced in various tropical and subtropical climes worldwide. It can help with diabetic treatment. The tree’s leaves contain bioactive substances, such as corosolic acid, which have been found to promote insulin function and increase glucose uptake by cells. It has excellent antioxidant properties due to many phenolic components, such as ellagitannins, flavonoids, and anthocyanins. Anti-inflammatory characteristics may help reduce inflammation and treat conditions, such as rheumatism and intestinal inflammation, as well as antibacterial activity. In traditional medicine, it is utilized for a range of additional uses. It is a diuretic, an antihypertensive, and an antipyretic. Furthermore, the tree’s bark, roots, and leaves are used to treat diarrhea, skin diseases, and respiratory problems [10,11].

Cancer is presently the most lethal disease characterized by unregulated cellular proliferation and growth, which poses a significant challenge when it metastasizes and becomes refractory to conventional therapeutic interventions [9]. Among the cancer types, osteosarcoma (OS) is a rare, orphan tumor of mesenchymal origin with multiple subtypes. OS, which mainly affects children and adolescents and has two peak incidents at the ages of 18 and 60, affects men more frequently than women. In 80% of cases, OS affects the limbs, particularly the long bones, due to the rapid bone growth and turnover during adolescence. The remaining events affect the axial skeleton and, in infrequent circumstances, the jaw or hand. Annually, 1–5 cases per million people are reported globally. These tumors represent less than 0.2% of all malignant tumors in the EUROCARE database [12,13].

Some researchers have also been employed to assess the efficacy of novel drugs for treating osteosarcoma and other conditions affecting the bones [14,15,16,17]. The bone osteosarcoma (MG-63) cell lines were significantly affected by green synthesized AgNPs. The ability of modified silver nanoparticle hydrogel coatings to inhibit the growth of microorganisms in vitro and their compatibility with MG-63 human osteoblast-like cells were both investigated by De-Giglio et al. [18]. Silver nanoparticles are synthesized from tridax plant leaves and mangrove plant leaf extract, and the application of AgNPs to osteosarcoma (MG-63) cell lines exhibits dose-dependent anti-metastatic, antimicrobial, and anticancer activity [19]. According to Singh et al. [20], seed extract mediates the biosynthesis of AgNPs, which has cytotoxic effects on osteosarcoma MG-63 cell lines, antioxidant activity, and antibacterial activity against broad clinical pathogens.

In addition, nanotechnology is still a good topic that can contribute significantly to innovation and advancement in the scientific community. The advancement of numerous fields and overcoming the difficulties posed by operating at the nano-scale will probably be driven by ongoing research in the future. Overall, the medicinal properties of the plant *L. speciosa* provide interest in the present investigation to synthesize silver nanoparticles.

Objectives of the research:

This research aims to synthesize AgNPs from *Lagerstroemia speciosa* flower bud extract, their characterizations using different analytical tools, and the evaluation of their biological activities. Therefore, the following objectives were carried out using *L. speciosa* flower bud extract:

(i) Bio-inspired synthesis of AgNPs utilizing the flower bud extract of the *L. speciosa* plant;
(ii) Characterization of biosynthesized AgNPs by using various analytical techniques;
(iii) Evaluating the antimicrobial activity of biosynthesized AgNPs against human clinical pathogens;
(iv) Determination of in vitro anticancer potential by MTT assay using biosynthesized AgNPs against MG-63 cell line and exploring the apoptotic inducing ability of biosynthesized AgNPs through flow cytometry.

## 2. Materials and Methods

### 2.1. Collection and Preparation of Flower Bud Extract

**Figure 1:**
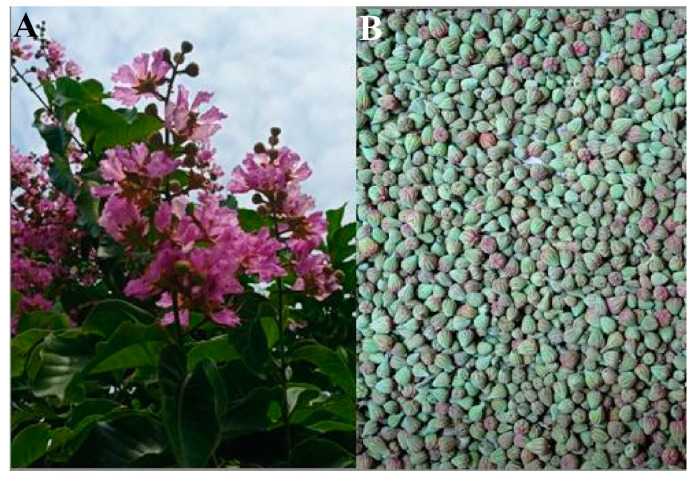
(**A**) *Lagerstroemia speciosa* plant habit and (**B**) collected flower buds.

*L. speciosa* flower buds were collected from the Botanical garden on the campus of Karnatak University, Dharwad, Karnataka, India (Latitude 15° 26′ 7″ N, Longitude 74° 58′ 57″ E) (Figure 1A,B). The flower buds were thoroughly washed, and shade dried at room temperature (26 °C) and then ground into a fine powder using an electric blender. The blended powder (5 g) was mixed with 100 mL of distilled water to prepare flower bud extract. The solution was filtered through Whatman No.1 filter paper after heating at 60 °C for 1 h and then cooled to room temperature. The filtrate was stored at 4 °C in a refrigerator for further analysis [21].

### 2.2. Synthesis of Silver Nanoparticles

Approximately 0.1689 g of AgNO_3_ was minced in 1000 mL of distilled water in an Erlenmeyer flask to obtain a 1 mM solution. Obtained flower bud extract (100 mL) was mixed with 1 mM AgNO_3_ solution (800 mL) in the ratio 1:8 (*v*/*v*) in a clean conical flask, and the pH was set to 9.0 and kept at room temperature in the dark for 24 h. The color of the reaction solution changed after the incubation period was the first hint of the fabrication of Ls-AgNPs. Ls-AgNPs were obtained from the reaction solution using subsequent centrifugation at 10,000 rpm for 20 min; then, air-dried and stored for further investigations [8].

### 2.3. Characterisation of Bio-Fabricated Ls-AgNPs

#### 2.3.1. UV-Visible Spectrophotometric Analysis

Using a UV-Visible spectrophotometer (UV-9600A, METASH, Shanghai, China), the spectroscopic analysis was utilized to monitor the formation of Ls-AgNPs. The reaction mixture was placed in a quartz cuvette, and the absorbance from 300 to 600 nm was measured. The graph was made by plotting wavelength against absorbance [22].

#### 2.3.2. Fourier Transform Infrared Spectroscopic Analysis

The detection of functional groups serving as stabilizing and capping agents during the synthesis of Ls-AgNPs was accomplished through FTIR analysis. The Ls-AgNPs were subjected to a drying process at 45 °C for a duration of 24 h in order to eliminate any water molecules present. Thin pellets were prepared by combining *L. speciosa* flower bud extract and Ls-AgNPs separately with 10% KBr. The spectra of the flower bud extract and Ls-AgNPs were recorded using an FTIR spectrophotometer (NICOLET-6700, Thermo Fischer Scientific, Waltham, MA, USA) over a wavelength range of 4000 to 400 cm^−1^ [23].

#### 2.3.3. X-ray Diffraction Analysis

XRD analysis was accomplished to determine the crystalline nature, phase variation, and grain size of Ls-AgNPs. The Ls-AgNPs were oven-dried at 50 °C and analyzed by an XRD analyzer (Rigaku Miniflex 600, smart-Lab SE, Tokyo, Japan) employing a Cu-K*α* radiation (λ = 1.54056 A°) source. The spectrum was recorded in the 2*θ* scanning range of 30° to 90°, and the spectrum was matched with the standard JCPDS file no: 04-0783 [24].

#### 2.3.4. Energy Dispersive X-ray Analysis

For this analysis, the Ls-AgNPs were utterly dried, and a small amount of sample was mounted on carbon tape and coated with gold sputtering for 2 min. It was then analyzed with the instrument (JEOL, JSM IT 500LA, Peabody, MA, USA) to identify the elemental makeup of Ls-AgNPs [25].

#### 2.3.5. Transmission Electron Microscopy

To figure out the size, shape, and structures of Ls-AgNPs, TEM (FEI, TECNAI G2, and F30, Beijing, China) analysis was performed. Five microliters of Ls-AgNPs were placed on the surface of the copper grid and dried in desiccation for 48 h. Later, the Ls-AgNPs were scanned at a voltage of 300 keV, and images were captured at the resolution of 7000× to 8000× [26].

#### 2.3.6. Zeta Potential and Dynamic Light Scattering Analysis

The surface charge of Ls-AgNPs was ascertained by zeta potential analysis in a colloidal system. A uniform suspension of nanoparticles was prepared by ultra-sonication and centrifuged at 6000 rpm for 20 min. It was then analyzed by a Nano analyzer (Horiba scientific nanoparticle analyzer SZ-100, Kyoto, Japan) at 3.4 eV. The dispersal type and average size of Ls-AgNPs were analyzed with DLS measurement [27].

#### 2.3.7. Thermo Gravimetric Analysis

The thermal properties and decomposition of Ls-AgNPs synthesized through the biological process were analyzed using a TGA instrument (SDT Q 600, New Castle, DE, USA). A predetermined amount of Ls-AgNPs was introduced into the furnace at ambient temperature (approximately 27 °C) and subjected to a gradual temperature increase up to 800 °C, with a heating rate of 10 °C/min, In contrast, inert nitrogen gas (at a flow rate of 40 mL/min) was passed over the sample, as described in references [28,29].

### 2.4. Antimicrobial Activity of Ls-AgNPs

The antimicrobial efficacy of Ls-AgNPs was determined by employing the agar well diffusion technique against four distinct microbial pathogens. The Gram-positive organism: *Staphylococcus aureus* (MTCC 6908), Gram-negative organism: *Escherichia coli* (MTCC 40), yeast: *Candida albicans* (MTCC 227) and *Candida glabrata* (MTCC 3019) were procured from IMTECH, Chandigarh, India. A suspension of Ls-AgNPs (1 mg/mL) was prepared using DMSO, and nutrient agar medium was utilized for antimicrobial activity. One hundred microliters of bacterial and yeast inoculums (0.5 McFarland concentrations) were swabbed on medium surfaces using sterile cotton swabs. Sterile cork borers were utilized to create wells measuring 6 mm in diameter. The wells were filled with varying volumes of Ls-AgNPs, precisely 25, 50, 75, and 100 µL. Distilled water served as the negative control; streptomycin and nystatin (1 mg/mL) were chosen as the standard reference for bacteria and yeast, respectively. After 18 h of incubation period at 37 °C, the diameter of the zone of inhibition surrounding each well was measured in millimeters (mm) [30,31].

### 2.5. In-Vitro Anticancer Activity of Ls-AgNPs

#### 2.5.1. MTT Assay

The MG-63 cell line, derived from human osteosarcoma, was procured from NCCS, Pune. The anti-proliferative assay was conducted using the MTT-96 well plate method. The cell line was subjected to sub-culturing in DMEM and added with FBS (10%). The cancer cells were maintained at 37 °C for 24 h in an atmosphere containing 5% CO_2_. The MTT assay was conducted using a negative control of medium and cells without Ls-AgNPs. Quercetin (30 µM/mL) was utilized as a positive control. MG-63 cells were seeded at a density of approximately 20,000 cells per well in 96-well plates and incubated for 24 h to allow for growth. Subsequently, cellular entities were subjected to a suspension of AgNPs, with concentrations varying from 12.5 to 200 µg/mL, along with the positive control, for 24 h. Following incubation, the medium was decanted and replaced with 200 µL of MTT reagent per well. The cells were then kept for 3 h of incubation. MTT-formazan crystals were dissolved by adding 100 µL of DMSO and gently shaking the mixture. The absorbance of the resulting suspension was recorded at 570 nm using an ELISA reader (ELX-800, BioTek, Winooski, VT, USA) [32,33], which marked the final step of the procedure. On the basis of the dose-response curve and the following formula, the concentration of Ls-AgNPs required to inhibit cell growth by 50% (IC_50_) was calculated: Y = Mx + c. The percentage of cell viability was determined using the below-following formula:

$$\%\;\mathrm{cell}\;\mathrm{viability}=\frac{\mathrm{Optical}\;\mathrm{density}\;\mathrm{value}\;\mathrm{of}\;\mathrm{treated}\;\mathrm{cells}}{\mathrm{Optical}\;\mathrm{density}\;\mathrm{value}\;\mathrm{of}\;\mathrm{untreated}\;\mathrm{cells}}\times 100$$

#### 2.5.2. Annexin-V/Propidium Iodide Apoptosis Detection Assay

The apoptotic effects of Ls-AgNPs on MG-63 cells were evaluated through the utilization of the FITC Annexin-V (51-65874X) and propidium iodide (PI) (51-66211E) labeling technique. A total of 20,000 MG-63 cancer cells were seeded into a 96-well plate and treated with Ls-AgNPs at the IC_50_ concentration of 37.57 µg/mL for 24 h under controlled conditions of 37 °C and 5% CO_2_. The cells without any treatment were designated as untreated control, while quercetin-treated control cells were designated as such. Further, the cancer cells were washed using PBS buffer and subjected to a 3–4 min treatment with 200 µL of trypsin-EDTA at 37 °C. Subsequently, a volume of 2 mL of culture medium was introduced, and the cells were subjected to centrifugation at 300 rpm for 5 min, after which the supernatant was carefully removed. Following that, PBS was used to wash the cells twice, after which they were subjected to staining with 5 µL of Annexin-V and incubated in a light-free environment for 15 min. In addition, a mixture comprising five µL of PI and 1X binding buffer (400 µL) was cautiously vortexed with the cells. Subsequently, the cells were subjected to analysis via a flow cytometer (BD FACS Calibur), and the investigation outcomes were computed utilizing BD Cell Quest Pro Ver.6.0 software [34].

### 2.6. Statistical Analysis

The experiments were conducted in triplicate (*n* = 3), and the mean of three parallel experiments used to determine each value. The presentation of each outcome was in the form of mean ± standard deviation (±SD). The obtained results were subjected to statistical analysis using SPSS and Origin 2022 Pro version.

## 3. Results

### 3.1. Synthesis of Silver Nanoparticles and UV-Visible Spectrophotometric Analysis

**Figure 2:**
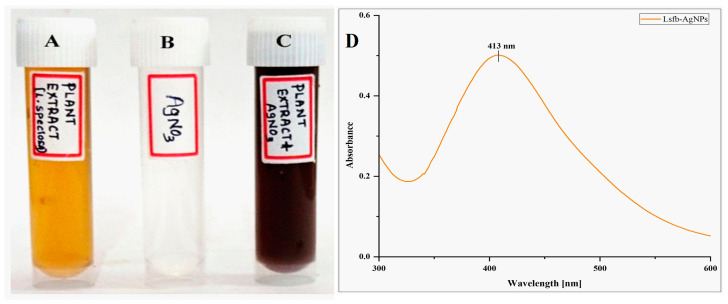
Synthesis of Ls-AgNPs from *L. speciosa*: (**A**) pale yellow colored flower bud aqueous extract, (**B**) 1 mM AgNO_3_ solution, (**C**) change of the color to darkish brown after an incubation period of 24 h, and (**D**) UV-Visible absorption spectrum of synthesized Ls-AgNPs.

The aqueous flower bud extract of Ls-AgNPs was minced with one mM silver nitrate (AgNO_3_) suspension in a ratio of 1:8 at pH 9.0 to synthesize Ls-AgNPs. After 24 h of the incubation period, the reaction mixture turned dark brownish from pale yellow, indicating proper synthesis and fabrication of Ls-AgNPs (Figure 2A–C). Due to phytochemicals’ reduction and capping action in the flower bud extract, silver ions (Ag^+^) were bio-reduced into elemental silver (Ag°) and capped and stabilized. Synthesized Ls-AgNPs produced a characteristic absorption spectrum at 413 nm, suggesting metallic silver’s surface plasmon resonance (SPR) range (Figure 2D).

### 3.2. FTIR Analysis

**Figure 3:**
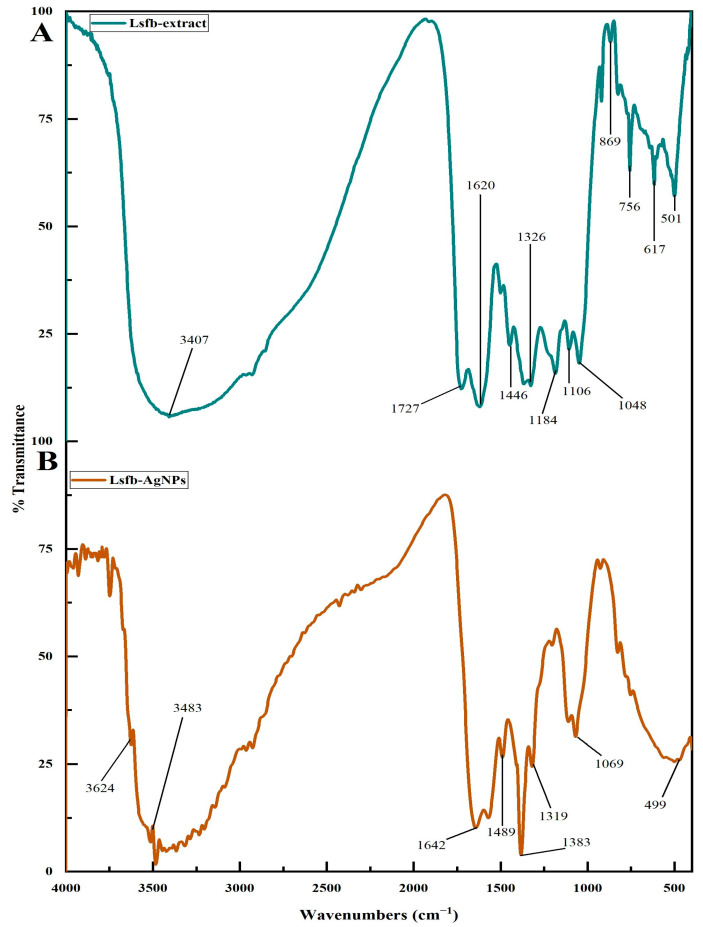
(**A**) FTIR spectrums of *L. speciosa* flower bud extract and (**B**) synthesized Ls-AgNPs showing the presence of functional groups.

The FTIR spectrum of biosynthesized Ls-AgNPs depicted 8 vibrational peaks compared to 12 in the *L. speciosa* flower bud extract in the region of 4000 to 400 cm^−1^ (Figure 3A,B). The spectral analysis of Ls-AgNPs and bud extract indicates a shift in the broad peak towards a higher wavelength, specifically from 3624 to 3407 cm^−1^, which can be attributed to the O–H stretching of alcohol. Additionally, the presence of N=H stretching primary amine is evidenced by the peak observed at 3483 cm^−1^ in the Ls-AgNPs spectrum. The observed displacement of the peak from 1727 to 1642 cm^−1^ indicates the existence of conjugated aldehyde C=O stretching. The peak shifting at 1620 to 1489 cm^−1^ represents the C=C stretching of conjugated alkenes and C=H bending alkane. The reallocation of the peak from 1446 to 1383 cm^−1^ corresponded to the C=H bending alkane and C=H bending aldehyde. The minimal displacement of the peak from 1319 cm to 1326 cm^−1^ indicates the presence of O–H bending phenol. Additionally, the vibrational peaks observed at 1184 and 1069 cm^−1^ in the flower bud extract correspond to the C–O stretching of aromatic esters and S=O stretching of sulfoxide compounds, respectively. The flower bud extract displayed a minor vibrational peak at 1048 cm^−1^, which was indicative of the C–N stretching of amines. Additionally, the vibrational peaks observed at 869, 756, and 617 cm^−1^ were attributed to the C=C bending of tri-substituted alkenes, C–Cl stretching of the halo compound, and C–Br stretching of the halo compound, respectively. The observed displacement of the peak towards a higher wavelength from 501 to 499 cm^−1^ has been attributed to the stretching of C–I bonds in halo compounds. The observed alterations in the peaks of the FTIR spectrum suggest an active role of the functional groups of phyto-constituents in synthesizing NPs from the extract of flower buds.

### 3.3. XRD Analysis

**Figure 4:**
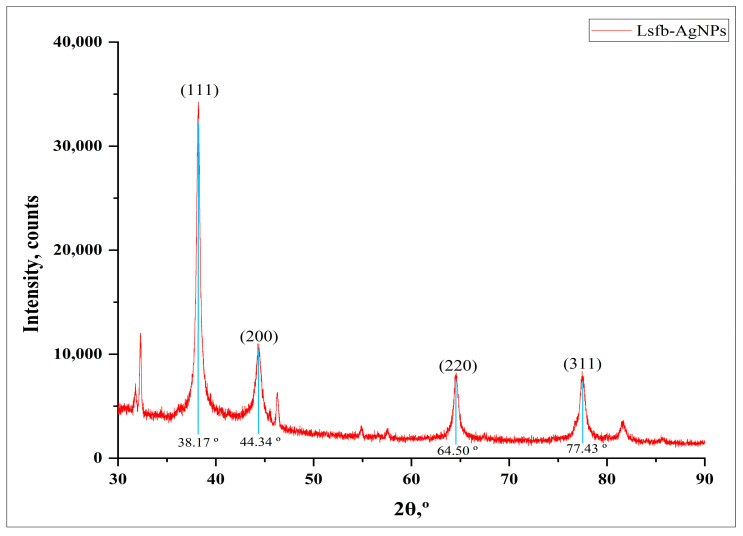
XRD pattern of biosynthesized Ls-AgNPs.

The crystalline nature of Ls-AgNPs was determined through X-ray diffraction analysis, which exhibited distinct Bragg’s reflection planes. The XRD pattern illustrates the primary reflection planes at 2*θ*, with corresponding values of 38.17°, 44.34°, 64.50°, and 77.43°, as shown in Figure 4. The XRD analysis revealed that the metallic silver possessed a face-centered cubic (FCC) crystalline nature, as evidenced by identifying the 111, 200, 220, and 311 facets of Bragg’s planes.

### 3.4. EDX Analysis

**Figure 5:**
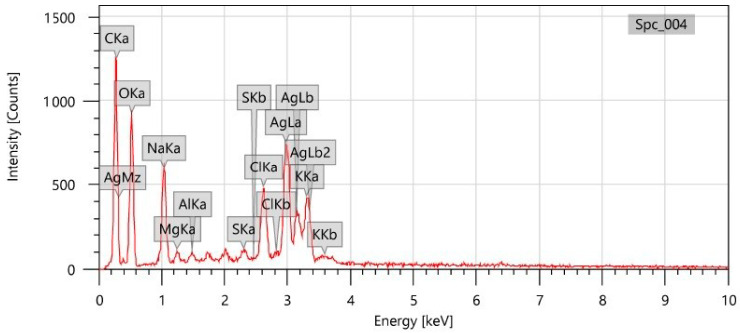
EDX spectrum of biosynthesized Ls-AgNPs.

The EDX analysis was conducted to assess the existence of diverse chemical elements implicated in the synthesis of Ls-AgNPs. The EDX spectrum was obtained by analyzing the dried powder form of Ls-AgNPs within the energy range of 0 to 10 KeV. The EDX spectrum displayed a discernible peak at 3 keV, a characteristic feature of pure metallic silver. Additionally, the spectra revealed the existence of 39.80% silver in the sample. In addition to the presence of Ag, discernible peaks indicated the existence of various other elements, such as carbon, oxygen, chlorine, sodium, potassium, and calcium, at distinct intervals (Figure 5). The potential existence of reported elements could be attributed to the accessibility of diverse phyto-constituents present in the flower bud extract of *L. speciosa*, which acts as reducing and capping agents.

### 3.5. TEM Analysis

**Figure 6:**
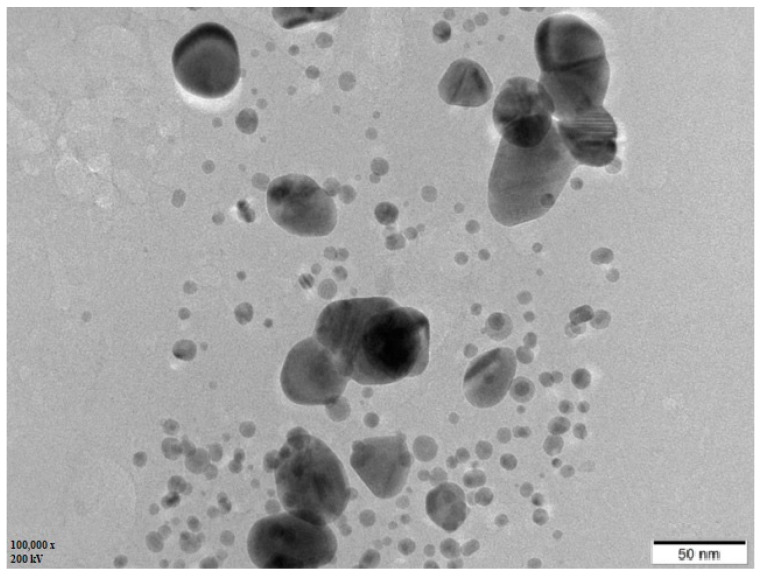
TEM image of biosynthesized Ls-AgNPs.

The TEM micrographs revealed a detailed analysis of the morphology, distribution, and size of Ls-AgNPs. Morphologically Ls-AgNPs were determined as spherical with little scattered aggregation and poly-dispersed. The size computed for Ls-AgNPs ranged from 10.27 to 62.5 nm (Figure 6).

### 3.6. Zeta Potential and DLS Analysis

**Figure 7:**
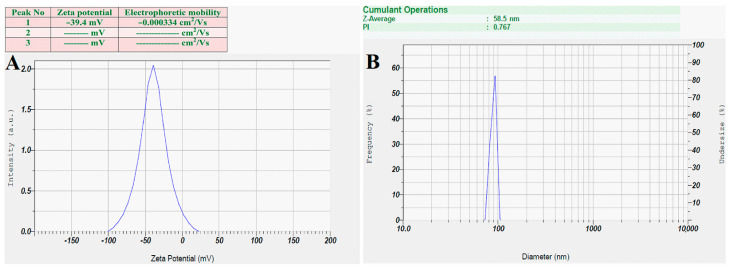
(**A**) Zeta potential analysis graph of biosynthesized Ls-AgNPs, and (**B**) DLS analysis graph of biosynthesized Ls-AgNPs.

The synthesized Ls-AgNPs were dispersed in an aqueous colloidal medium at ambient temperature, and the resulting zeta potential was determined to be −39.4 mV (Figure 7A). The Ls-AgNPs synthesized through biosynthesis were observed to possess a negative zeta potential. This observation suggests a repulsive force among the silver nanoparticles that were synthesized through green synthesis, which ultimately leads to an increase in their stability. The high negative zeta potential of silver nanoparticles results in their poly-dispersed nature. The electrostatic repulsion between nanoparticles impedes their agglomeration, thereby promoting term stability. The dynamic light scattering technique is commonly employed to ascertain silver nanoparticles’ dimensions and dispersion characteristics of AgNPs. The mean size of Ls-AgNPs was determined to be 58.5 nm, as illustrated in Figure 7B.

### 3.7. TGA Analysis

**Figure 8:**
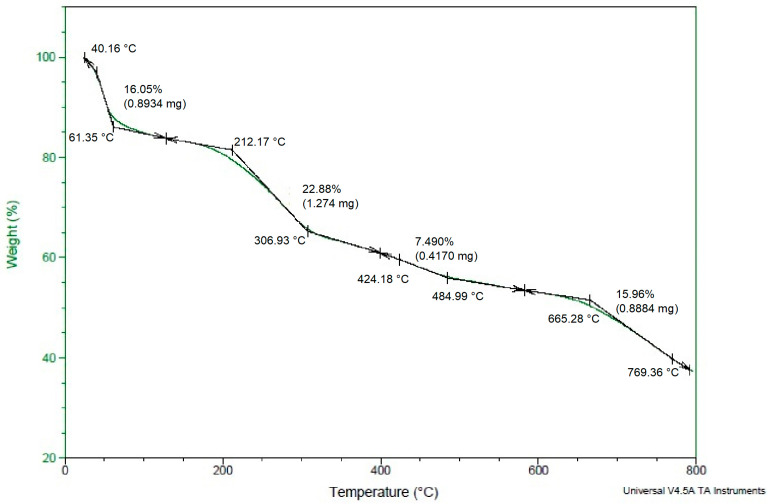
Thermo gravimetric analysis curve of biosynthesized Ls-AgNPs.

The thermal stability of the biosynthesized Ls-AgNPs was determined through a TGA plot, as depicted in Figure 8. The TGA curve demonstrated a high level of stability within the temperature range of 27 °C to 800 °C, with minimal weight loss. The thermal degradation behavior of Ls-AgNPs was investigated, and it was observed that three significant weight losses of 22.88%, 7.49%, and 15.96% occurred as the temperature was increased from 40 °C to 306 °C, 307 °C to 484 °C, and 485 °C to 769 °C, respectively. The initial loss in weight can be attributed to the process of moisture evaporation. In addition, the subsequent two weight losses are primarily attributable to the desorption of active bio-organic phyto-constituents that act as conjugated bio-molecules on the surface of AgNPs.

### 3.8. Antimicrobial Activity of Synthesized Ls-AgNPs

**Figure 9:**
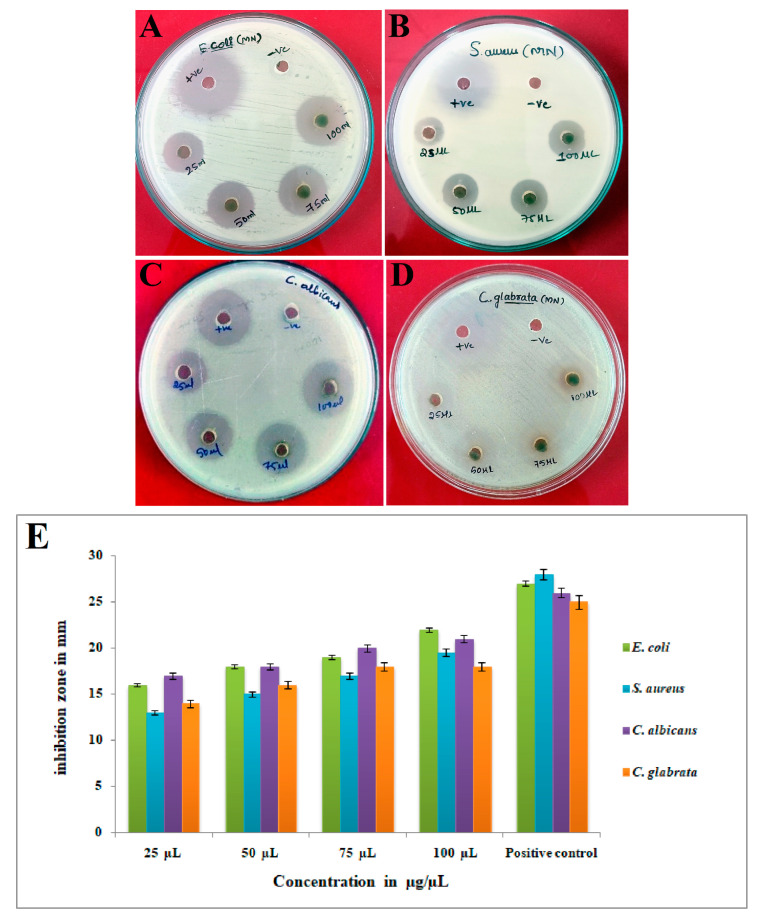
Antimicrobial activity of Ls-AgNPs biosynthesized from the flower buds extract (**A**) *E.coli*, (**B**) *S. aureus*, (**C**) *C. albicans*, (**D**) *C. glabrata*, and (**E**) graph showing the zone of inhibition for Ls-AgNPs.

The findings of the antimicrobial activity demonstrated significant efficacy against all examined clinical pathogens. The figure denoted as Figure 9A–D illustrates the antimicrobial properties of Ls-AgNPs. Figure 9E presents a graphical depiction of the inhibition zones resulting from various concentrations of Ls-AgNPs, positive control. The findings elucidated that the inhibitory effect underwent a gradual modification with the escalation in the concentration of AgNPs. The results indicate that E. coli and C. albicans exhibited the highest susceptibility to the maximum concentration of Ls-AgNPs (100 µL), with inhibition zones of 22 mm and 19.5 mm, respectively. Conversely, S. aureus and C. glabrata demonstrated the lowest susceptibility, with 18 mm and 18.5 mm inhibition zones, respectively.

### 3.9. Anticancer Activity of Silver Nanoparticles by MTT Assay

**Figure 10:**
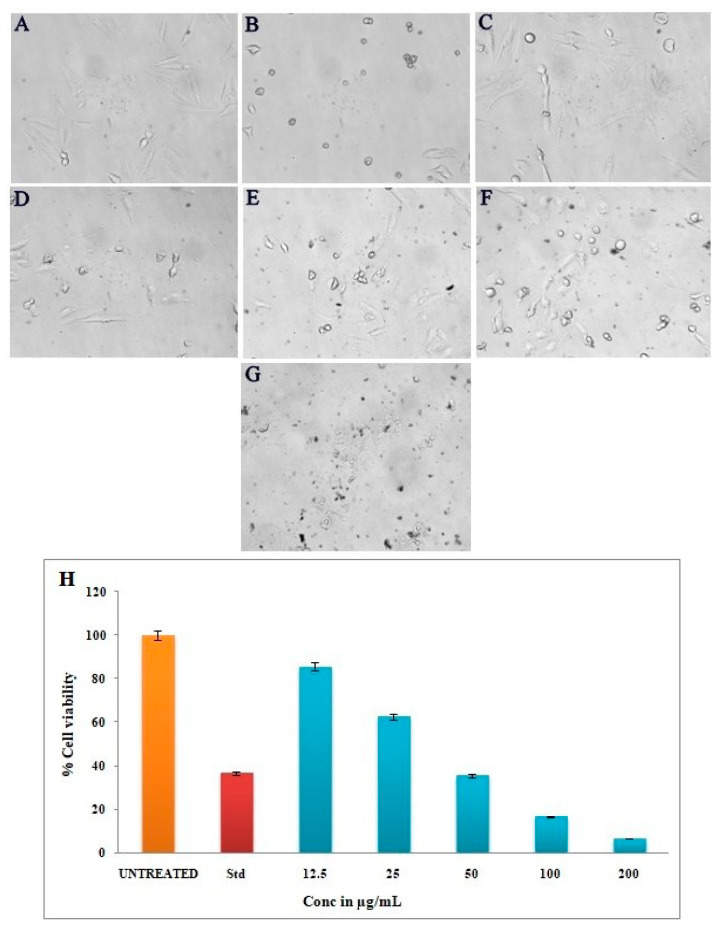
MTT assay of different volumes of biosynthesized Ls-AgNPs: (**A**) untreated cells, (**B**) standard control, (**C**) 12.5 μg/mL, (**D**) 25 μg/mL, (**E**) 50 μg/mL, (**F**) 100 μg/mL, (**G**) 200 μg/mL, and (**H**) bar graph showing comparative cell viability percentages.

The biosynthesized Ls-AgNPs were found to result in a direct and dose-dependent cytotoxic effect on MG-63 cells, as depicted in Figure 10C–G. Figure 10A,B depicts the negative and positive controls, respectively. The cytotoxicity exhibited a gradual increase with the gradual rise in the concentration of Ls-AgNPs, ranging from 12.5 to 200 µg/mL. The percentage of viable cancer cells observed was 85.78%, 62.74%, 35.76%, 16.88%, and 6.87%, respectively. The IC_50_ value was determined to be 37.57 µg/mL.

### 3.10. Apoptosis/Necrosis Assay by Flow Cytometry

**Figure 11:**
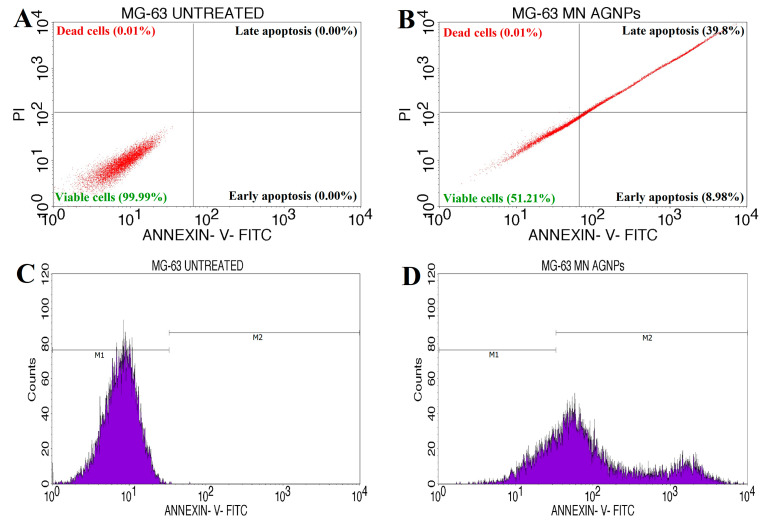
Quadrangular plots showing the Annexin V/PI expression in MG-63 cancer cells: (**A**) untreated cells, (**B**) cancer cells treated with Ls-AgNPs, (**C**) cell cycle arrest of untreated cells, and (**D**) cell cycle arrest of treated cells.

A test was performed to assess the occurrence of apoptosis/necrosis in the MG-63 cell line following treatment with a synthesized Ls-AgNPs solution at the IC_50_ concentration. The aim was to determine the extent of early and late apoptosis. The neoplastic cells were subjected to FITC Annexin V/PI staining, and the determination of early and late apoptotic cells was validated via flow cytometry using fluorescent activated cell sorting (FACS), as depicted in Figure 11A,B. The study revealed that the induction of apoptosis in cancer cells was observed upon treatment with Ls-AgNPs at an IC_50_ concentration of 37.57 µg/mL for a duration of 24 h. The treated cells exhibited a substantial increase in the population of early apoptosis cells (8.98%) and late apoptosis cells (39.8%), while the untreated cells did not exhibit any significant apoptosis. Figure 11C,D depicts the analysis of cell cycle arrest during the M1 and M2 phases. The results indicate that the cells not subjected to any treatment exhibited a viability of 99.95% for M1 and 0.05% for M2, as illustrated in Figure 11C. In Figure 11D, it can be observed that M1 accounted for 25.28% of the viable MG-63 cancer cells, while M2 comprised 74.72% of the damaged cells.

## 4. Discussion

The present research reported the nano-bio-fabrication of silver nanoparticles using *L. speciosa* flower bud extract with the determination of antimicrobial and anticancer activity. In the recent evolution of nanotechnology, many approaches were looked into synthesizing AgNPs from different parts of plant-like roots, leaves, stems, flowers, fruits, etc. The initial hint of Ls-AgNPs biosynthesis was assured by a color change in the reaction solution of flower bud extract and AgNO_3_ (1:8 *v*/*v*). The color change from pale yellowish to dark brownish indicated the surface plasmon resonance of metallic silver, which in turn attributed to the biosynthesis of Ls-AgNPs. During the synthesis, various phyto-constituents in the flower bud extract were directly proportional to the synthesis of Ls-AgNPs due to their reducing and capping activity [35,36]. The UV absorbance peak for Ls-AgNPs was at 413 nm, and the absorption band was due to the surface plasmon resonance of nanoparticles. Similar observations reported the role of *R. serrata* flower bud extracts containing various phytochemicals in the reduction, capping, and stabilization of AgNPs [37].

The molecular atmosphere on the surface of Ls-AgNPs due to the capping of *L. speciosa* phytoconstituents was investigated by FTIR analysis. The shifting of peaks in the Ls-AgNPs FTIR spectrum was owing to the interaction between several chemical compounds and Ag^+^ [38]. The *L. speciosa* flower bud extract was encapsulated by phytochemicals and some acids due to functional groups, including alkanes, alkenes, amines, alcohols, aldehydes, ketones, etc. The functional groups on Ls-AgNPs showed the existence of primary and secondary amines, indicating that proteins were involved in the reduction and stabilization of Ag^+^. Thus, the AgNPs were reduced and stabilized by the *L. speciosa* flower extract’s nano-capping. This result was agreed with the report of Rajesh Kumar et al. [39], where the AgNPs synthesized from *C. guianensis* Aubl., bud extract expressed the presence of several functional groups.

The face-centered cubic crystalline nature of Ls-AgNPs was affirmed by X-ray diffractometry. The XRD pattern showed the reflection peaks at 38.17°, 44.34°, 64.50°, and 77.43° at 2θ degrees corresponding to the 111, 200, 220, and 311 facets of Bragg’s planes. Accordingly, a previous study involved in the synthesis of AgNPs from *Cucumis sativus* var. *hardwickii* exhibited a similar XRD pattern, having Bragg’s reflection indexes of 111, 200, 220, and 311 at 2*θ* angles of 38.25°, 46.41°, 64.55°, and 77.48°. According to the previous report, the additional peaks that were acquired could potentially be attributed to alternative crystalline phases associated with the inorganic components of the plant extract, which are present on the surface of the synthesized silver nanoparticles [40].

The EDX spectrum of Ls-AgNPs exhibited 39.80% of Ag in the sample. This also demonstrated the reduction of Ag^+^ ions to Ag^o^ by generating a major peak at 3 keV. The EDX spectrum also revealed the existence of other metals, such as carbon, chlorine, sodium, potassium, etc. Similarly, the AgNPs obtained from *Syzygium aromaticum* (Clove) extract demonstrated a strong signal for Ag with other peaks for calcium, carbon, oxygen, chlorine, potassium, etc. The elemental peaks, apart from Ag, were observed due to the presence of bio-molecules on the surface of AgNPs involved in the capping and stabilization; the peaks for carbon and oxygen might be due to the plant materials and atmospheric moisture [41].

The TEM micrograph illustrated the shape of Ls-AgNPs as spherical and poly-dispersed with slight agglomerations at some points. The particle sizes were assessed in the range of 10.27 to 62.5 nm. Similar observations were recorded in previous studies, where the AgNPs synthesized from clove buds extract showed spherical-shaped, poly-dispersed AgNPs in size ranges of 10 to 50 nm. The report suggested that most AgNPs were aggregated, while a few were scattered with varied sizes [42]. In other investigations, it was reported that the AgNPs from *C. guianensis* Aubl. bud extract were spherical, 5 to 40 nm in size, and poly-dispersed [39].

Zeta potential exhibits the nanoparticles’ dispersion stability and surface electric charge. The zeta analysis of Ls-AgNPs showed a sharp peak at −39.4 mV, indicating an anionic surface charge and moderate repulsion between AgNPs. The zeta potential value indicated moderate stability. Similarly, El-Aswar et al. [43] reported that the zeta value of the biosynthesized silver nanoparticles from an *H. tuberculatum* extract was −42.6 mV, suggesting higher repulsion among AgNPs and their dispersion stability. According to previous studies, zeta potential higher than +30 mV or −30 mV was strongly cationic and strongly anionic; the more considerable negative/positive zeta potential value indicated higher repulsion among AgNPs, leading to higher stability [41].

The DLS analysis determined the size distribution profile of Ls-AgNPs and calculated the average size of particles as 58.5 nm. The prominent DLS spectrum was a sign of proper synthesis of nanoparticles. Brownian motion of nanoparticles in the suspension was the key to measuring the size of Ls-AgNPs. These results were in accordance with the previous report suggesting the calculated average size of *P. murex* leaf extract AgNPs at 73.14 nm [44].

The TGA analysis illustrated the thermal stability of Ls-AgNPs by depicting the % weight loss at a range of temperatures from room temperature to 800 °C. Initially, at room temperature, the weight of Ls-AgNPs was 100%, gradually decreasing to 55% at 700 °C with a total weight loss of 46.25%. These results were compared with TGA results of synthesized NPs from *C. pulcherrima* aqueous flower-extract, where the AgNPs weight loss was observed constantly from 0 to 800 °C, and the total weight loss up to the end (800 °C) for the biosynthesized silver nanoparticles is nearly 71.68% [45].

The synthesized green Ls-AgNPs demonstrated antimicrobial activity that was dependent on the dosage. The findings of the current investigation indicate that *S. aureus* exhibited greater susceptibility towards Ls-AgNPs, while *E. coli* demonstrated the highest resistance level compared to the other microbial pathogens selected. Singh [46] reported comparable findings, whereby AgNPs derived from smaller-sized NPs demonstrated heightened sensitivity towards *P. aeruginosa, E. coli*, *K. pneumonia*, and *B. subtilis.* The findings indicate that the size of the dose administered may influence the efficacy of AgNPs against multidrug-resistant bacteria. Lee et al. [47] elucidated in a prior investigation that the utilization of *Tussilago farfara* buds extracts for producing of AgNPs resulted in distinctive inhibition of the growth of various microbial strains. The fascinating antimicrobial efficacy can be attributed to the proportion of AgNPs’ extensive surface area to their volume, facilitating a more robust interaction with pathogens. The notable impact of AgNPs on microbial pathogens can be attributed to their diminutive size and the electrostatic force of attraction between the less negative to the positive charge of the nanoparticles and the negatively charged membrane of the microbial cells. The conversion of chemical and physiological properties has been observed to disrupt their normal physiological functions, such as permeability and respiration [48,49]. Silver nanoparticles have been found to induce the production of reactive oxygen and nitrogen species, resulting in oxidative stress on DNA and other cellular components. This disturbance of cellular functions ultimately leads to bacterial cell death, as reported in previous studies [50].

**Figure 12:**
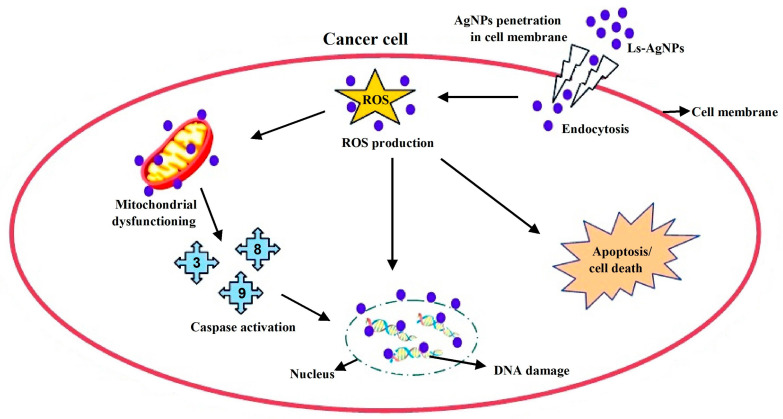
Schematic representation of possible model mechanism and mode of action of silver nanoparticles in cancer cells.

The Ls-AgNPs that were bio-fabricated demonstrated remarkable anti-cancer properties on the MG-63 cell line, with the effect being dependent on the dosage administered. The experiment results demonstrate that the application of escalating concentrations of Ls-AgNPs (ranging from 12.5 to 200 µg/mL) to cancer cells resulted in a notable reduction in viable cells, decreasing from 85.78% to 6.87%. The untreated control group exhibited complete cell viability, while the standard group demonstrated cell viability of 30.92%. The IC_50_ value of Ls-AgNPs was determined to be 37.57 µg/mL, indicating a promising outcome. An array of literature is available documenting the anti-proliferative properties of silver nanoparticles against diverse cancer cell lines. Figure 12 illustrates the possible mechanism that may account for the cytotoxic effects of AgNPs on cancer cell lines. According to existing literature, the cytotoxicity of AgNPs is primarily attributed to the induction of oxidative stress and apoptosis through a caspase-dependent mechanism, leading to DNA impairment, mitochondrial malfunction, and eventual cell death [8,37]. Nithya et al. [14] reported a comparable outcome, whereby the utilization of biosynthesized AgNPs from *Vitrus vinifera* fruit extract resulted in the inhibition of MG-63 cell proliferation. The observed cell inhibition was 55% at a concentration of 1.5 mM of AgNPs. According to Nayak et al. [17], silver nanoparticles are currently utilized in bone cementing materials and prosthetic devices; these facilitate rapid recovery and exhibit potential therapeutic benefits for breast cancer, skin cancer, and wound healing. The biosynthesized Ls-AgNPs exhibited significant overall anticancer activity compared to previous studies on MG-63 cell inhibition techniques.

The Annexin V-FITC/PI apoptosis method was utilized to verify the occurrence of apoptosis. The Ls-AgNPs exhibited a notable ability to induce apoptosis against the MG-63 cell line, with an IC_50_ value of 37.57 µg/mL. The primary mechanism of numerous anticancer drugs is to induce cancer cell death by triggering the apoptotic signaling pathways [51]. Comparably, Niu et al. [52] found that the apoptotic effects on MG-63 cells were similar between DOX/MLT-loaded nano-carrier treatment and free DOX/MLT treatment for 24 h. The nano-carrier induced 28.78% and 3.05% early apoptosis and 28.7% and 50.4% late apoptosis, respectively. A study by Iram et al. [53] revealed that the administering of Tb_2_O_3_ nanoparticles at a concentration of 0.102 μg/mL resulted in an inevitable outcome. The quantification of cells with compromised membrane integrity was performed using PI/FITC uptake. The MG-63 cells subjected to Tb_2_O_3_ NPs exhibited a concentration-dependent uptake of PI/FITC and demonstrated early apoptosis rates of 5.3% and 6.4%, respectively. The study utilizing Annexin V-FITC/PI aimed to identify cells undergoing early and late stages of apoptosis. According to a previous study [54], cells were deemed viable if they exhibited negative Annexin V and PI results. In contrast, cells indicating apoptosis or necrosis were identified as positive for Annexin V and PI. An elevated concentration of silver ion has the potential to induce oxidative stress, thereby resulting in an escalation of reactive oxygen species and consequent cytotoxicity [40]. The observed cytotoxicity can be attributed to the effective interaction between AgNPs and the phosphate groups in cellular structures’ DNA, nitrogen bases, and protein functional groups. According to previous research, the activation of reactive oxygen species due to the presence of silver nanoparticles can harm cellular components, ultimately culminating in cell death [55,56].

## 5. Conclusions

The present investigation delineated a facile, sustainable, and economical approach for the bio-fabrication of AgNPs utilizing the extract of *L. speciosa* flower buds. The confirmation of AgNP formation is supported by the UV-Vis spectrum, which displays a distinctive peak at 413 nm. The crystallinity of Ls-AgNPs was confirmed to be of the FCC type through XRD analysis. The stability of the Ls-AgNPs was demonstrated by the notable zeta potential value, while the average size of the particles was determined to be 58.5 nm through DLS analysis. The analysis conducted using TEM confirmed the presence of spherical particles that were randomly distributed, while the thermal stability of Ls-AgNPs was established through TGA analysis. Comprehending the influence of nanoparticles’ physical and chemical properties on their biological effects is of utmost importance. The Ls-AgNPs that were synthesized exhibit antimicrobial properties against various pathogens and demonstrate anticancer activity against the MG-63 cell line. Moreover, the flow cytometry technique was employed to evaluate the apoptosis/necrosis of neoplastic cells. The Annexin-V-FITC assay was employed to verify the induction of apoptosis initiation by Ls-AgNPs. This assay is widely utilized to distinguish living cells from those undergoing early and late apoptosis. The discernible actions exhibited by Ls-AgNPs are of significant importance in the advancement of novel therapeutic agents for the diagnosis of cancer. The employment of plant-mediated synthesis is deemed more advantageous compared to other methods due to its faster and more stable rate of metal ion reduction. Silver nanoparticles exhibit promising potential in biomedical applications, particularly in disease diagnosis, cellular and deep tissue imaging, drug/gene delivery, and multi-functional therapies.

## Data Availability

All data generated or analyzed during this study are included in this published manuscript.
